# Evidence of disparities in the provision of the maternal postpartum 6-week check in primary care in England, 2015–2018: an observational study using the Clinical Practice Research Datalink (CPRD)

**DOI:** 10.1136/jech-2021-216640

**Published:** 2021-09-09

**Authors:** Yangmei Li, Jennifer J Kurinczuk, Christopher Gale, Dimitrios Siassakos, Claire Carson

**Affiliations:** 1 NIHR Policy Research Unit in Maternal and Neonatal Health and Care, National Perinatal Epidemiology Unit, Nuffield Department of Population Health, University of Oxford, Oxford, UK; 2 Neonatal Medicine, School of Public Health, Faculty of Medicine, Chelsea and Westminster Hospital Campus, Imperial College London, London, UK; 3 Institute for Women’s Health, University College London, London, UK; 4 University College London Hospitals NIHR Biomedical Research Centre, London, UK; 5 Wellcome/EPSRC Centre for Interventional and Surgical Sciences (WEISS), London, UK

**Keywords:** cohort studies, health services, maternal health, perinatal epidemiology, primary care

## Abstract

**Background:**

A maternal postpartum 6-week check (SWC) with a general practitioner (GP) is now considered an essential service in England, a recent policy change intended to improve women’s health. We aimed to provide an up-to-date snapshot of the prevalence of SWC prior to the policy change as a baseline, and to explore factors associated with having a late or no check.

**Methods:**

We conducted a cohort study using primary care records in England (Clinical Practice Research Datalink (CPRD)). 34 337 women who gave birth between 1 July 2015 and 30 June 2018 and had ≥12 weeks of follow-up post partum were identified in the CPRD Pregnancy Register. The proportion who had evidence of an SWC with a GP was calculated, and regression analysis was used to assess the association between women’s characteristics and risks of a late or no check.

**Results:**

Sixty-two per cent (95% CI 58% to 67%) of women had an SWC recorded at their GP practice within 12 weeks post partum, another 27% had other consultations. Forty per cent had an SWC at the recommended 6–8 weeks, 2% earlier and 20% later. A late or no check was more common among younger women, mothers of preterm babies or those registered in more deprived areas.

**Conclusions:**

Nearly 40% of women did not have a postpartum SWC recorded. Provision or uptake was not equitable; younger women and those in more deprived areas were less likely to have a record of such check, suggesting postpartum care in general practice may be missing some women who need it most.

## Introduction

In February 2020, it was announced that a standardised postpartum check for women at 6–8 weeks after giving birth would be included as an essential service in the general practitioner (GP) contract, supported by £12 million additional funding.^1^ This is a change of direction after a series of policy decisions that have seen the GPs’ role in maternity care in England diminish over the past 20 years.

Per-capita ‘item of service’ payments for maternity services were removed from the 2004 GP contract when payments were rolled into the global sum paid to GPs, reducing the financial incentive to provide care during and after pregnancy.^2 3^ Policy decisions to give women direct access to midwives and to move midwives into Children’s Centres further distanced GP practices from maternity care. Yet, it has remained a recommendation that both women and their babies have a postnatal check with their GP at 6–8 weeks post partum,^4 5^ and the National Maternity Review *Better Births* report highlighted the importance of this ‘Six Week Check’ (SWC) in monitoring the health and well-being of both the mother and the baby.^6^ Although evidence on benefits of a maternal SWC has been sparse,^7^ the National Institute for Health and Care Excellence recommends that the maternal SWC should focus on: mental health and general well-being; return to physical health and identification of pelvic health issues; family planning and contraception; and pregnancy-related or birth-related conditions requiring ongoing management.^4 5^

The Chief Medical Officer (CMO) 2014 Annual Report stated that in 2008–2014, 73% of women received an SWC from their GP,^2^ with another study reporting only 56.2% of women in the UK had a structured postnatal check documented between 2006 and 2016.^8^ Ongoing changes in GP provision since then may have adversely affected this coverage. We aimed to describe women’s interactions with GPs in the first 12 weeks post partum, including the number and timing of SWCs, to provide an up-to-date snapshot of the prevalence of SWC in England in the period preceding the recent policy change, as a baseline for any future assessments for coverage of the SWC. Specifically, we aimed to identify the proportion of women who had the recommended SWC and the characteristics associated with a delayed check or not having an SWC.

## Methods

### Data source

Primary care data for England were drawn from the Clinical Practice Research Datalink (CPRD) using the Pregnancy Register to identify eligible women.^9 10^ The CPRD GOLD data set, on which the Pregnancy Register is based, currently covers around 4.7% of the UK population,^11^ and is broadly representative in terms of age, sex and ethnicity.^9^ The methods for the creation of the Pregnancy Register are described in detail elsewhere and validation studies show good concordance with hospital delivery data.^10^ In brief, the register is algorithm based using antenatal, birth and postnatal events in both mother and child primary care records (where available) to determine pregnancy episodes and derive dates for pregnancy trimesters and outcome, including an estimated date of birth.

### Study population

A total of 38 601 women aged 11–48 were identified who had a live birth or stillbirth between 1 July 2015 and 30 June 2018 and had ≥12 weeks of follow-up post partum. Pregnancies ending in miscarriage, termination or with an unknown outcome have been excluded. To provide the most up-to-date picture, where women had more than one birth in the period, we included the most recent as we aimed to estimate recent prevalence. The Pregnancy Register algorithm uses postnatal records to define or adjust the birth date in some cases. We excluded pregnancies that used postnatal records to define or adjust the birth date (n=4164, 11%), and a further 100 women (0.3%) who had multiple SWC records which complicated an analysis of timing, leaving 34 337 women for analysis (‘study population’).

No patients were involved in setting the research question, nor in the design, conduct or interpretation of the study. The study is based on anonymised national routine primary care data, and no dissemination of results directly to study participants is planned. However, the project had arisen from the National Maternity Review,^6^ which had involved consultation with women and provides background patient and public involvement for the study. In addition, the Expert Reference Group convened by the National Health Service (NHS) England that agreed the recommended content of a maternal GP check (which informs Read codes of interest in this study) had representation from service users and a range of patient representative groups.

### Deriving SWC variables

Relevant records of face-to-face consultations and examinations within 12 weeks post partum were identified. Where women had no recorded events, we considered there was no face-to-face interaction with the practice. Evidence of consultations related to SWC was identified using the recorded ‘medcodes’ (online supplemental table S1) and data entered under the maternity-related structured data area in the GP software. Women with codes specifically describing maternal SWCs were considered as having an SWC; women with other codes indicating a possible SWC, for example, ‘postnatal examination observations’, were considered as having an SWC if the relevant events happened between 4 and 12 weeks post partum. The SWCs were grouped into early checks before 6 weeks, at the recommended 6–8 weeks, 1 week late and 2 or more weeks late.

10.1136/jech-2021-216640.supp1 Supplementary data

### Other variables

Maternal age was derived using year and month of birth (where available), and the baby’s date of birth, and grouped into 5-year age bands. Mother’s ethnicity was identified as per Mathur *et al*,^12^ checked against published code lists^13^ and then categorised (White British; White Other; Asian or Asian British; Black or Black British; Mixed or Other). Preterm birth (<37 weeks of gestation) is provided as part of the Pregnancy Register based on records of gestational age or algorithm-estimated length of pregnancy. Geographical region of the practice, and area deprivation scores of both the mother’s area of residence and practice’s location as coded according to the English Index of Multiple Deprivation (IMD) were linked by CPRD.

### Analysis

The number and proportion of women giving birth who had evidence of an SWC were calculated and key characteristics of the study population described. As the outcome of interest (no SWC) is relatively common, a multivariable modified Poisson regression model was used to estimate the relative risk,^14^ including the characteristics listed above to further elucidate the association between women’s characteristics and whether they had an SWC.

The characteristics of groups who had an SWC early, on time (6–8 weeks), 1 week late or later were further compared. As GP practices differ in size, the population served and culture, CIs and p values were estimated accounting for clustering by GP practice, unless otherwise stated. Women with missing data were described as a separate group and automatically removed from the Poisson regression model. Stata V.15.1 was used for all analyses.

### Sensitivity analyses

The CPRD defines practices that reach certain reporting targets as ‘up to standard’.^9^ Sensitivity analyses were conducted by restricting the population to women who gave birth after the ‘up to standard’ date for the reporting practice. We also assessed the effect of excluding the women where postpartum records were used to define pregnancy end date by analysing all women (n=38 601).

## Results

### The population

Table 1 shows the characteristics of the 34 337 women included in the analysis. The largest proportion of mothers were aged 30–34 years (33%); 3% of mothers were under 20, and 5% aged 40 or older. For the 76% of the women whose ethnicity data were available, approximately 20% were from black, Asian and minority ethnic (BAME) groups. The geographical distribution of the sample favoured the South East Coast (22%) and London (22%), with fewer women from other parts of England. Area deprivation scores for the study population were slightly more affluent than the national average.

**Table 1:** The study population: most recent births to women in CPRD Pregnancy Register in England, July 2015 to June 2018 (n=34 337)

Characteristic	GP appointments in the first 12 weeks post partum*						Total	%	National figures for all births for comparison (%)†
	Women who had a maternal 6*-week check*		Women who had other GP appointments		Women who had no GP appointment				
	n	%	n	%	n	%			
Number of women in the sample	21 460	100	9149	100	3728	100	34 337	100	
Maternal age, mean (95% CI)	30.9 (30.6 to 31.2)		30.1 (29.8 to 30.5)		29.8 (29.5 to 30.1)		30.6 (30.3 to 30.9)		30.3 to 30.5
<20 years	440	2	295	3	125	3	860	3	3
20–24 years	2403	11	1303	14	607	16	4313	13	14
25–29 years	5428	25	2530	28	1040	28	8998	26	28
30–34 years	7303	34	2845	31	1138	31	11 286	33	32
35–39 years	4751	22	1730	19	657	18	7138	21	18
≥40 years	1135	5	446	5	161	4	1742	5	4
Preterm birth (<37 weeks of gestation)	1345	6	866	9	317	8.5	2528	7	8
Ethnic group									
White British	10 620	64	4683	68	1616	62	16 919	65	69
White Other	2646	16	956	14	427	16	4029	15	8
Asian or Asian British	1456	9	569	8	245	9	2270	9	9
Black or Black British	941	6	313	5	171	7	1425	5	5
Mixed or Other	880	5	342	5	159	6	1381	5	10
*Missing*	*4917*	*23*	*2286*	*25*	*1110*	*30*	*8313*	*24*	*6*
Geographical region									
North East, Yorkshire and Humber	482	2	265	3	90	2	837	2	14
North West	1986	9	1608	18	789	21	4383	13	13
Midlands	2523	12	1191	13	404	11	4118	12	11
East of England	1574	7	601	7	174	5	2349	7	11
South West	2137	10	1007	11	182	5	3326	10	9
South Central	3175	15	1024	11	241	6	4440	13	N/A
London	5068	24	1560	17	731	20	7359	21	19
South East Coast	4515	21	1893	21	1117	30	7525	22	15
Individual-level area deprivation (IMD)									
1 (least)	4248	25	1628	21	429	14	6305	23	15
2	3220	19	1365	18	507	16	5092	18	17
3	3218	19	1469	19	562	18	5249	19	19
4	3401	20	1575	21	768	25	5744	21	23
5 (most)	3130	18	1550	20	809	26	5489	20	27
*Missing*	*4243*	*20*	*1562*	*17*	*653*	*18*	*6458*	*19*	–
Practice-level area deprivation (IMD)									
1 (least)	4244	20	1264	14	276	7	5784	17	15
2	3834	18	1741	19	628	17	6203	18	17
3	4394	20	2019	22	887	24	7300	21	19
4	3659	17	1752	19	834	22	6245	18	23
5 (most)	5329	25	2373	26	1103	30	8805	26	27
*Missing*	*0*	*0*	*0*	*0*	*0*	*0*	*0*	*0*	–

Proportions are presented as the % of non-missing, unless in italics, where % is of all women. North East and Yorkshire and Humber are combined due to small numbers.

*Face-to-face appointments in a GP practice.

†Comparison figures drawn from: Office for National Statistics (ONS)^28^ for mean maternal age in 2015–2017; ONS^29^ for maternal age groups in 2017 births; Draper *et al* 30 for preterm birth in 2016; ONS^15^ for births by geographical area and IMD group in 2017; Li *et al* 16 for births by maternal ethnicity (2006–2012).

CPRD, Clinical Practice Research Datalink; GP, general practitioner; IMD, Index of Multiple Deprivation; N/A, not applicable.

### Evidence of an SWC

Sixty-two per cent (95% CI 58% to 67%) of women had records indicating an SWC in the first 12 weeks post partum, and a further 27% had one or more consultations not classified as an SWC. Approximately 11% of the women had no evidence of face-to-face contact with their GP during this period. Table 2 shows the relative risk of not having an SWC, by women’s characteristics. Younger women, those who gave birth preterm and those cared for by practices serving more deprived areas were less likely to have evidence of an SWC.

**Table 2:** Crude and adjusted relative risk of not having clear evidence of a maternal 6-week check in the first 12 weeks post partum, by population characteristics (n=34 337)

Characteristic	Women who had a maternal 6-week check in the first 12 weeks post partum n (row %)	Women who had no clear evidence of a maternal 6-week check in the first 12 weeks post partum n (row %)	Unadjusted relative risk (95% CI)*	Fully adjusted relative risk (95% CI)*
Number of women	21 460 (62)	12 877 (38)		
Maternal age				
<20 years	440 (51)	420 (49)	1.38 (1.26 to 1.53)†	1.32 (1.22 to 1.43)†
20–24 years	2403 (56)	1910 (44)	1.25 (1.17 to 1.35)†	1.21 (1.14 to 1.28)†
25–29 years	5428 (60)	3570 (40)	1.12 (1.07 to 1.18)†	1.10 (1.06 to 1.14)†
30–34 years	7303 (65)	3983 (35)	Reference group	Reference group
35–39 years	4751 (67)	2387 (33)	0.95 (0.91 to 0.99)†	0.96 (0.92 to 1.01)
≥40 years	1135 (65)	607 (35)	0.99 (0.92 to 1.06)	1.00 (0.93 to 1.07)
Preterm birth, n (%)	1345 (53)	1183 (47)	1.27 (1.16 to 1.39)†	1.26 (1.15 to 1.37)†
Practice-level area deprivation (IMD)				
1 (least deprived)	4244 (73)	1540 (27)	Reference group	Reference group
2	3834 (62)	2369 (38)	1.43 (0.95 to 2.16)	1.41 (0.94 to 2.11)
3	4394 (60)	2906 (40)	1.50 (1.02 to 2.20)†	1.45 (0.98 to 2.13)
4	3659 (59)	2586 (41)	1.56 (1.07 to 2.26)†	1.49 (1.03 to 2.16)†
5 (most deprived)	5329 (61)	3476 (39)	1.48 (1.01 to 2.18)†	1.40 (0.95 to 2.07)

*CIs and p values account for clustering by GP practice; variables included in the fully adjusted model were maternal age, preterm birth and practice-level IMD.

†P<0.05.

GP, general practitioner; IMD, Index of Multiple Deprivation.

### Timing of the SWC

**Figure 1:**
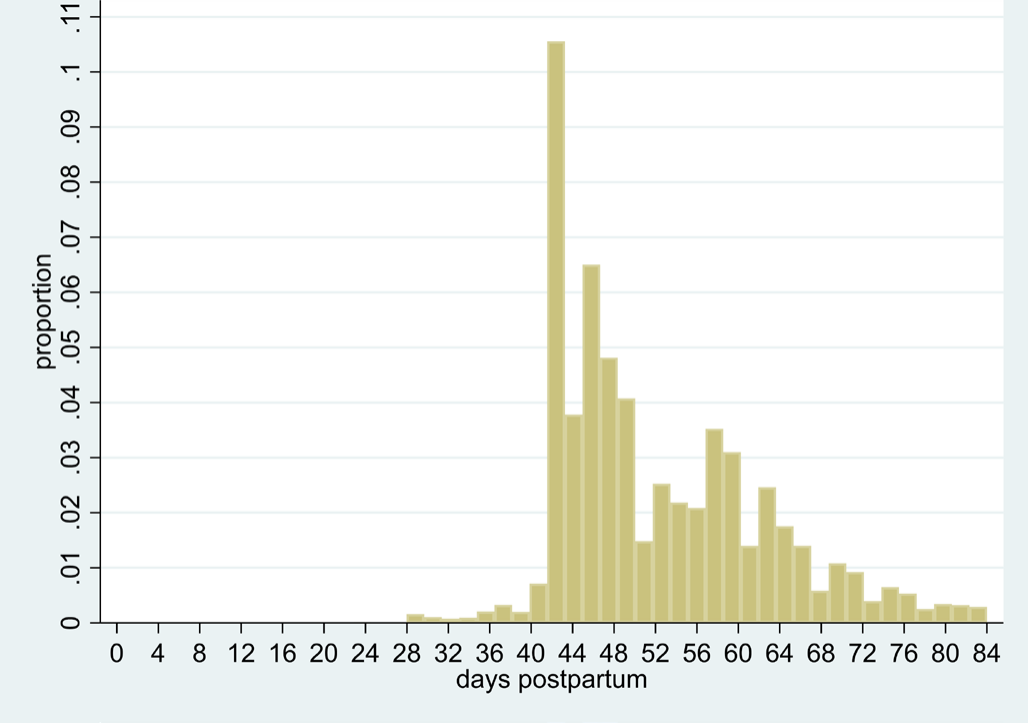
Distribution of identified maternal 6-week checks in the first 12 weeks post partum, over time (n=34 337).

Figure 1 presents the distribution of SWCs over the 12 weeks after giving birth, showing a peak at 6–8 weeks (42–56 days) and another 2 weeks later. Table 3 shows the characteristics of the women according to the timing of her SWC. The proportion of women having SWC on time was higher in women aged ≥30 (approximately 44%) than in younger women (33%–38%), while the proportion of women who had a late check or no check was higher in younger women. Those who experienced a preterm birth were less likely to have a timely SWC (30% vs 41%) and more likely to have a late check (11% vs 9%) or no check (47% vs 37%) compared with women who had a term birth. Compared with white women, BAME women were less likely to have had an SWC on time (36% vs 43%) but the overall proportion of women who had not had an SWC was similar. The temporal pattern of SWC varied by geographical region: while over 40% of the mothers in East of England, South East, London and South West had an SWC at 6–8 weeks, a quarter or fewer had a check at the recommended time in the North West, or North East and Yorkshire and Humber. Around 57% of women served by practices in the least deprived IMD quintile had a timely SWC compared with 33% of those in the most deprived quintile.

**Table 3:** Variation in timing of the maternal 6-week check in the first 12 weeks post partum, by maternal characteristics (n=34 337)

Characteristic		Timing of the 6-week check, n (row %)					
		Early (<6 weeks)	Recommended (6–8 weeks)	1 week late (9 weeks)	≥2 weeks late (10–12 weeks)	Not at all	Total
Overall		697 (2)	13 843 (40)	3818 (11)	3102 (9)	12 877 (38)	34 337 (100)
Within age groups	<20 years	9 (1)	254 (30)	84 (10)	93 (11)	420 (49)	860 (100)
	20–24 years	70 (2)	1428 (33)	461 (11)	444 (10)	1910 (44)	4313 (100)
	25–29 years	172 (2)	3375 (38)	1017 (11)	864 (10)	3570 (40)	8998 (100)
	30–34 years	231 (2)	4904 (43)	1209 (11)	959 (9)	3983 (35)	11 286 (100)
	35–39 years	179 (3)	3111 (44)	828 (12)	633 (9)	2387 (33)	7138 (100)
	≥40 years	36 (2)	771 (44)	219 (13)	109 (6)	607 (35)	1742 (100)
Preterm birth	No	660 (2)	13 081 (41)	3551 (11)	2823 (9)	11 694 (37)	31 809 (100)
	Yes	37 (1)	762 (30)	267 (11)	279 (11)	1183 (47)	2528 (100)
By ethnic group	White	425 (2)	8982 (43)	2185 (10)	1674 (8)	7682 (37)	20 948 (100)
	BAME	123 (2)	1831 (36)	700 (14)	623 (12)	1799 (35)	5076 (100)
	*Missing*	149 (2)	3030 (36)	933 (11)	805 (10)	3396 (41)	8313 (100)
Within geographical regions	East of England	X*	1148 (49)	223 (9)	X*	775 (33)	2349 (100)
	London	189 (3)	3105 (42)	1043 (14)	731 (10)	2291 (31)	7359 (100)
	Midlands	96 (2)	1503 (37)	519 (13)	405 (10)	1595 (39)	4118 (100)
	North East, Yorkshire and Humber	X*	214 (26)	170 (20)	X*	355 (42)	837 (100)
	North West	116 (3)	949 (22)	418 (10)	503 (11)	2397 (55)	4383 (100)
	South East	198 (2)	5574 (47)	1070 (9)	848 (7)	4275 (36)	11 965 (100)
	South West	63 (2)	1350 (41)	375 (11)	349 (10)	1189 (36)	3326 (100)
By individual-level area deprivation (IMD)	1 (least deprived)	143 (2)	3074 (49)	622 (10)	409 (6)	2057 (33)	6305 (100)
	2	106 (2)	2196 (43)	539 (11)	379 (7)	1872 (37)	5092 (100)
	3	96 (2)	2104 (40)	536 (10)	482 (9)	2031 (39)	5249 (100)
	4	108 (2)	1966 (34)	702 (12)	625 (11)	2343 (41)	5744 (100)
	5 (most deprived)	98 (2)	1705 (31)	712 (13)	615 (11)	2359 (43)	5489 (100)
	*Missing*	146 (2)	2798 (43)	707 (11)	592 (9)	2215 (34)	6458 (100)
By practice-level area deprivation (IMD)	1 (least deprived)	104 (2)	3295 (57)	547 (9)	298 (5)	1540 (27)	5784 (100)
	2	166 (3)	2281 (37)	760 (12)	627 (10)	2369 (38)	6203 (100)
	3	100 (1)	2963 (41)	696 (10)	635 (9)	2906 (40)	7300 (100)
	4	169 (3)	2365 (38)	589 (9)	536 (9)	2586 (41)	6245 (100)
	5 (most deprived)	158 (2)	2939 (33)	1226 (14)	1006 (11)	3476 (39)	8805 (100)

North East and Yorkshire and Humber are combined due to small numbers.

*Cells are suppressed because of small numbers in the cells.

BAME, black, Asian and minority ethnic; IMD, Index of Multiple Deprivation.

### Sensitivity analyses

Restriction to practices considered ‘up to standard’ at the time of the birth (n=33 099) showed little effect on the prevalence: 20 729 (63%) had an SWC. The women excluded to allow accurate assessment of the timing of the check (n=4264) were slightly older, more affluent and more likely to be from London and South East Coast (online supplemental table S2). When they were also included (total n=38 601), 65% (95% CI 61% to 69%) of women had records indicating an SWC in the first 12 weeks post partum.

## Discussion

### Main findings

In this analysis of recent routine primary care data for England, 89% of women saw a GP in the first 12 weeks post partum. Sixty-two per cent of women had an SWC recorded, and a further 27% had consultations not classified as an SWC. Overall, 40% of women had an SWC at the recommended 6–8 weeks. Younger women, women who gave birth preterm or who were served by practices in more deprived areas were more likely to have a late or no SWC.

### Strengths and limitations

The strengths of the study include the use of the Pregnancy Register, the most recently available routine primary care data and a comprehensive code list to identify maternal SWCs. This study captured approximately 2% of all maternities in England during the study period; where women had more than one pregnancy during the study period we included the most recent birth to provide the most up-to-date picture of postpartum checks in primary care. While the CPRD is considered broadly representative of the UK in terms of age, sex and ethnicity,^9^ the geographical distribution of births in these data favoured London and the South East.^15^ The CPRD Pregnancy Register is algorithm based; restriction to women where postpartum records were not used to define or adjust the birth date is a cautious approach that gives a slightly more conservative SWC rate. The estimated prevalence of an SWC in the ‘full population’ is not substantially different from the restricted ‘study population’, 65% (95% CI 61% to 69%) vs 62% (95% CI 58% to 67%). By excluding this group, the association between age, deprivation and the risk of not having an SWC is also likely to be more conservative.

Some factors in this study may result in an overestimate of the prevalence. Our analysis indicates affluent and older mothers, who are over-represented in the data set, are more likely to have an SWC. The data included a slightly higher proportion of white non-British mothers compared with national estimates for 2006–2012.^16^ This is plausible, however, as the proportion of births to mothers from other European countries has risen from 9% to 13% of live births between 2012 and 2017.^17^ White non-British mothers may be more likely to attend an SWC, as the majority are from other European countries^18^ where maternity care tends to be more medicalised.^19^

There are also some plausible influences that may lead to an underestimate of the true population prevalence. This study focuses on SWCs delivered by the GP, therefore our figures are based on primary care data and do not include the women having SWCs in hospitals. Some of the consultations and examinations not classified as an SWC involved postpartum issues covered in a recommended SWC, such as contraception advice and postnatal depression. These may have been an SWC where the GP only recorded the main issues discussed or problems identified; as we used a more stringent definition for SWC, they were not classified as such.

Information regarding behavioural factors that may affect the uptake of the SWC, such as education level and family support, could not be assessed using routine primary care data. Exploring the association between these factors and SWC would require primary data collection or qualitative research. Parity was not available in this study so we used only the most recent pregnancy for each woman to provide the most up-to-date snapshot of the prevalence of SWCs.

While the results of our study are applicable to the population of England, the international generalisability may be limited by differences in compositions and characteristics of women giving birth, structure of postnatal care and also access to this care.

### Interpretation

Our estimates are lower than those in the 2014 CMO report, where 73.5% of mothers between 2008 and 2014 were reported to have had a postpartum check.^2^ These differences may be partly due to differing methods for identifying pregnancies, and a stricter definition of the SWC in the current analysis as we excluded codes indicating early postpartum contacts or individual postpartum issues. However, it is plausible that fewer SWCs actually happened in the period of the current analysis. Data from the 2018 National Maternity Survey found 91% of women reported a check of their own health with their GP.^20^ This is almost certainly an overestimate of those receiving an SWC covering all the recommended aspects, as it is very close to our figure of 89% for any GP consultation across the 12-week period. Moreover, survey responders might have been more proactive in managing their health, including requesting and attending an SWC. A study from the UK found 56.2% of women had a structured postnatal check documented between 2006 and 2016.^8^ The slightly lower prevalence may reflect an even more stringent definition of SWC and shorter time window (weeks 5–10 post partum) but is largely consistent with our finding.

Although 89% of women in the present study had at least one face-to-face consultation at their GP practice within 12 weeks post partum, 27% of women had only consultations not classified as an SWC. It is possible that some GPs did not correctly record the check due to the lack of guidance of standardised recording and heavy workload. It is also possible that women seeing the GP for specific postpartum conditions did not subsequently attend an SWC, missing a comprehensive assessment and discussion of their postpartum health.

Our findings suggest some inequity in the provision or uptake of postpartum care as younger women and those served by practices in more deprived areas were more likely to have a late SWC or no check. This is consistent with literature reporting inequalities in maternal health^21^ and seems unsurprising given the mixed messages women receive; while the NHS website outlines what ‘should’ be part of the maternal SWC,^22^ it also says that ‘*Some GP surgeries do not routinely offer a postnatal check. You can always request an appointment for a check, especially if you have any concerns*’, thus placing the onus on the woman to seek care. Differences by age and area deprivation may also be due to variation in practices offering the SWC as routine, or in women’s own awareness of the checks. Women who gave birth preterm were more likely to have a late check or no check than those who had a term birth. This may be because they had spent longer in hospital themselves, or with their baby, but it also reflects a group of high-risk women who may be missing adequate postpartum care. Some of these women may have attended a hospital follow-up for debriefing, but this would not normally be as comprehensive as a GP SWC.

A 2014 National Childbirth Trust/Netmums survey of new mothers highlighted inconsistencies in the checks conducted, a lack of clarity among mothers about what to expect and a sense that checks were rushed.^23^ The recommended SWC covers a comprehensive list of issues, so is likely to take longer than usual appointments. This has implications for GPs, particularly where time and staff are already stretched. However, a comprehensive SWC offers an opportunity to identify and intervene across the spectrum of postpartum health problems, both mental and physical, with important long-term implications.

Following the announcement in February 2020 that a universal maternal SWC should be considered an ‘essential service’ in the GP contract,^1^ this study can now form a basis to monitor whether this change in policy has led to an increase in the coverage of SWC. Recent contractual changes should also help to address the observed inadequate provision or uptake in younger women and those living in the most deprived areas, but must be supported by clear, standardised guidance for GPs and training where needed. The recent changes to the contract also provide opportunities to better structure the GP recording system so that comprehensive checks like SWC can be recorded in a standardised way that indicates whether the check is provided and reflects results for individual aspects of the check. During the COVID-19 pandemic, the guidance recommends the maternal SWC is offered via remote consultation since face-to-face consultations must be minimised.^24^ It is important to monitor how the provision changes as the situation evolves, and assess its impact on women’s health.

We have identified younger maternal age, area deprivation and having a preterm birth as associated with not having an SWC or having a late SWC. Future studies exploring other factors that may potentially affect whether a woman receives an SWC, for example, educational level, parity and pre-existing conditions,^25 26^ may help further identify those who are at higher risk. There is sparse qualitative evidence on women’s experiences of postpartum care, especially for the general population.^27^ Further qualitative research focusing on experiences of SWC may help understand and improve women’s experiences. It would also be of interest to compare the health outcomes of women receiving and not receiving an SWC, and to assess outcomes of women whose condition was identified in an SWC, to quantify the benefits of a standardised universal SWC at both individual and national levels.

In conclusion, using recent national data, we present an up-to-date snapshot of mothers’ postnatal SWCs in primary care in England. Approximately 89% of women had a face-to-face consultation in a GP surgery by the end of 12 weeks post partum, and nearly two-thirds of women have a record of a recommended SWC in that time period. However, provision or uptake is not equitable, with younger women and those living in the most deprived areas most likely to miss out.

**What is already known on this subject**

- A National Health Service England Expert Reference Group on postnatal care and the National Institute for Health and Care Excellence both recommend a standardised health check for new mothers conducted by their general practitioner (GP) at 6–8 weeks after birth.
- Unlike the postnatal check for infants, until February 2020 there has been no contractual requirement for GPs to conduct a maternal postnatal check at 6–8 weeks.
- Reported coverage varies by source from 56.2% to 91%, and a current snapshot of the GP provision of maternal postnatal checks prior to the recent policy change is needed as a baseline for any future assessments.

**What this study adds**

- Eighty-nine per cent of women had had a face-to-face consultation with a GP in the 12 weeks after they gave birth and 62% of women had a consultation that could be clearly defined as a six-week postnatal check.
- Younger women, women who experienced a preterm birth or who were served by practices in more deprived areas were more likely to have a late or no six-week postnatal check, suggesting that postnatal care in general practice may be missing the women who need it most.

## Data Availability

Data may be obtained from a third party and are not publicly available. The data that support the findings of this study are available from Clinical Practice Research Datalink (CPRD). Restrictions apply to the availability of these data, which were used under licence for this study. The data were provided by the CPRD under a contractual agreement that does not permit the sharing of data. Study documentation is available on request from the corresponding author.
