# Supplementary material for: Evidence of disparities in the provision of the maternal postpartum 6-week check in primary care in England, 2015–2018: an observational study using the Clinical Practice Research Datalink (CPRD)
Source: J Epidemiol Community Health. 2021 Sep 9;76(3):239–46. doi: 10.1136/jech-2021-216640 (PMC8862061; doi:10.1136/jech-2021-216640)
Supplement: Supplementary data [file jech-2021-216640supp001.pdf]

Supplementary Table S1: Code list used to derive the Six Week Check variable

The following codes were used to derive the SWC variable, in combination with the event date.

Read Code	CPRD Medcode	Description
**Evidence of a Check**		
62S..00	11289	Maternal P/N 6 week exam.
62SZ.00	14706	Maternal P/N 6 week exam. NOS
62S..11	5071	Postnatal exam. - maternal
62S5.00	15429	Maternal P/N exam. done
62S6.00	29287	Postnatal examination minor problem found
62S7.00	4180	Postnatal examination normal
62Q2.00	29452	P/N care from G.P.
62Q6.00	19544	Postnatal care
62QZ.00	15672	Postnatal care NOS
62R..00	13585	Postnatal visits
62R..11	100418	Postnatal visit
62R..12	100230	New birth visit
62RZ.00	13587	Postnatal visit NOS
6G...00	12773	Postnatal care
9537.00	64738	Complete post-natal care
953D.00	53027	Full post-natal examination
9NV0.00	94071	Postnatal clinic
9N05.00	21345	Seen in postnatal clinic
9N1y400	36000	Seen in postnatal clinic
Z29..00	18458	Postnatal examination observations
ZV24.11	6217	[V]Postnatal care and examination
**Entity Type (from the administrative data area), used to identify SWC**		
	**Entity Code**	**Description**
	69	Post-natal examination
	150	Post-natal visit

Supplementary Table S2: Characteristics of the study population, excluded women and the full population

**Characteristic**	**Study population**		**Excluded women**		**Full population**		**National figures of all births for comparison**
	**N**	**%**	**N**	**%**	**N**	**%**	**%^**
	**34,337**		**4,264**		**38, 601**	**100**	
**Maternal age,** mean (95%CI)	30.6 (30.3-30.9)	-	31.1 (30.8-31.5)	-	30.7 (30.4-30.9)		
<20 years	860	3	72	2	932	2	3
20-24 years	4,313	13	466	11	4,779	12	14
25-29 years	8,998	26	1,054	25	10,052	26	28
30-34 years	11,286	33	1,474	35	12,760	33	32
35-39 years	7,138	21	956	22	8,094	21	18
≥40 years	1,742	5	242	6	1,984	5	4
**Preterm birth** (<37week gestation)	2,528	7	289	7	2,817	7	8
**Ethnic group**							
White British	16,919	65	2,085	65	19,004	65	69
White Other	4,029	15	464	14	4,493	15	8
Asian or Asian British	2,270	9	274	8	2,544	9	9
Black or Black British	1,425	5	229	7	1,654	6	5
Mixed or Other	1,381	5	177	5	1558	5	10
missing	*8,313*	*24*	*1,035*	*24*	*9,348*	*24*	*6*
**Geographical region**							
North East, Yorkshire & Humber	837	2	79	2	916	2	14
North West	4,383	13	333	8	4,716	12	13
Midlands	4,118	12	371	9	4,489	12	11
East of England	2,349	7	260	6	2,609	7	11
South West	3,326	10	320	8	3,646	9	9
South Central	4,440	13	471	11	4,911	13	?
London	7,359	21	1,290	30	8,649	22	19
South East Coast	7,525	22	1,140	27	8,665	22	15
**Individual-level area deprivation (IMD)**							
1 (least)	6,305	23	820	25	7,125	23	15
2	5,092	18	665	20	5,757	18	17
3	5,249	19	640	19	5,889	19	19
4	5,744	21	720	22	6,464	21	23
5 (most)	5,489	20	502	15	5,991	19	27

Characteristic	Study population		Excluded women		Full population		National figures of all births for comparison
missing	*6,458*	*19*	*917*	*22*	*7375*	*19*	-
**Practice-level area deprivation (IMD)**							
1 (least)	5,784	17	819	19	6,603	17	15
2	6,203	18	834	20	7,037	18	17
3	7,300	21	700	16	8,000	21	19
4	6,245	18	953	22	7,198	19	23
5 (most)	8,805	26	958	22	9,763	25	27
missing	*0*	*0*	*0*	*0*	*0*		-

^ Comparison figures drawn from: for mean maternal age in 2015-2017, from ONS (2019);[1] for maternal age groups in 2017 births, from ONS (2018);[2] for preterm birth in 2016, from Draper *et al* (2018);[3] for births by geographical area and IMD group in 2017, from ONS (2019);[4] for births by maternal ethnicity (2006-2012), from Li *et al* (2018).[5]

References

1. *Office for National Statistics. Birth characteristics in England and Wales: 2017.* 2019.
2. Office for National Statistics. Datasets: Births in England and Wales: summary tables. 2018.
3. Draper E, Gallimore I, Kurinczuk J, Smith P, Boby T, Smith L, et al. *MBRRACE-UK Perinatal Mortality Surveillance Report, UK Perinatal Deaths for Births from January to December 2016.* 2018.
4. Office for National Statistics. Figures on births by gestation, ethnic group, IMD and area of usual residence. 2017.
5. Li Y, Quigley MA, Dattani N, Gray R, Jayaweera H, Kurinczuk JJ, et al. The contribution of gestational age, area deprivation and mother's country of birth to ethnic variations in infant mortality in England and Wales: A national cohort study using routinely collected data. *PLoS One*. 2018;**13**(4):e0195146.
